# Reflections on the battle against COVID-19: The effects of emotional design factors on the communication of audio-visual art

**DOI:** 10.3389/fpsyg.2022.1032808

**Published:** 2022-11-07

**Authors:** Wen-Ting Fang, Jian-Hua Sun, Qing-Dong Liang

**Affiliations:** ^1^School of Art and Design, Shanghai Dianji University, Shanghai, China; ^2^Office of the CPC Shandong University Committee, Shandong University, Jinan, China; ^3^School of Education, Jiangsu University of Technology, Changzhou, China

**Keywords:** COVID-19, emotional design, emotional experience, audio-visual arts, communication effects

## Abstract

Fighting against the epidemic is an arduous and prolonged battle where many artists hope to inspire people with the power of art through cultural creativity. To explore the effects of emotional design factors on the communication of audio-visual art and the audience’s perceptive experience, this research takes the original anti-epidemic song and the film *China Braves Headwind* as the research object. The research also uses such methods as questionnaires, Structural Equation Models, and dependent samples *t*-tests to conduct statistical analysis. The results are as follows: First, the emotional design evaluation matrix based on the emotional communication model is reasonable, and the scales of this research are feasible. Second, the emotional design of audio-visual works can significantly affect the audience’s emotional experience and further improve sharing intention. Third, Attribute A2 (Artistic style, Thematic perception) and attribute C3 (Emotional resonance, Spiritual sublimation) serve as common factors affecting the emotional experience in terms of both musical works and film and television works. Fourth, compared with musical works, film and television works are likely to resonate with the audience. The combination of music and visual sensation can help open up the conception of artistic works and convey their meanings to viewers. Therefore, it’s necessary to explore the emotional communication mode between audio-visual artists and the audience. It helps artists think about how to create works innovatively and is conducive to marketizing works and stimulating cultural consumption demand.

## Introduction

As many countries around the world went into a frenzy of cultural creativity at the beginning of the 21st century, humanistic aesthetics has been placed high on the agenda in the design community. Developing an “aesthetic economy” with cultural characteristics has been an inevitable trend. In terms of creative cultural design, the key lies in extracting cultural elements and fostering a taste of lifestyle to finally transfer a wholly new aesthetic meaning to the audience through emotional content (Hsu and Lin, 2011; Yeh et al., 2011; Yang et al., 2022). Norman (2004) pointed out that emotional factors were the key to designing. A successful cultural and creative product can definitely arouse the inner desire of its audience. A high-quality design thus must be creative, culture-rooted, and human nature-centered from stem to stern.

The COVID-19 pandemic at the start of 2020 has brought heavy damage to countries worldwide in the aspect of economy, politics, and culture (World Health Organization, 2020). Individuals with a high fear of COVID-19 may experience negative emotions and cognitions (Brooks et al., 2020; Rettie and Daniels, 2020). Meanwhile, fighting against the epidemic is an arduous and prolonged battle where many artists hope to inspire people with the power of art through cultural creativity (Larissa et al., 2021; Lydia, 2022). The studies suggest a potential role for mixed emotions in pandemic-related outcomes, which can promote complex thought processes and eudaimonic well-being (Berrios et al., 2018; Oh and Tong, 2021). Some findings emphasize the importance of emotional experience and emotion regulation for self-efficacy, subjective wellbeing, and positive coping during the pandemic (Cattelino et al., 2021; İme and Ümmet, 2022). The results showed optimism as a protective factor against the psychological impact of the COVID-19 pandemic which can increase confidence, motivate individuals to achieve goals, and increase positive affect and well-being through its effects on perceived stress and infection stress anticipation (Brosschot et al., 2005; Puig-Perez et al., 2022). Art language is composed of codes, which is a well-organized and understandable information system. When artists have the same code system and code perception as their audience, the truth that their artistic creations can be deeply perceived makes art an emotion transmitter without frontiers. Philosopher Dewey (1964) also put forward that in a shared world full of divides and walls that limit people’s experience, artistic works serve as the only means of communication between people, completely without barriers. As a branch of the cultural and creative industries, audio-visual art has a unique form of artistic expression and is also an important way of emotional expression. Art creators, through their understanding, design situations and artistic conceptions for emotional communication. Artists with creative ideas intrinsically create artistic works, by which their desires and fantasies can be realized. In turn, the audience who enjoy these works can release their inner depression and can feel a sense of pleasure (Winner, 1982). The dissemination of art can relieve stress and negative emotions, which has a significant effect on fighting against COVID-19 (Lydia, 2022; Niels et al., 2022). Therefore, it is an issue worth exploring how artists express their creative ideas and inner feelings, and how viewers can grasp the essence of creativity and gain emotional experience during the COVID-19 pandemic.

During the epidemic, China’s economy has been hit severely, and the people’s normal life and production have been disrupted. The COVID-19 pandemic has caused new patterns of behaviors that differ from previous ones in terms of responses and emotions to external stimuli (D’Uggento et al., 2022). Online access to cultural activities could sustain the educational and entertaining demands of diverse groups during mass confinement (Samaroudi et al., 2020; Ginzarly and Srourb, 2022). Cultural and artistic activities online, such as virtual museums, virtual Concerts, art galleries, and live theaters, created more opportunities for people to experience the arts and achieve artistic consumption (Choi et al., 2020). Studies found a remarkable increase in the consumption of digital cultural offerings (e.g., music, film, literature, and theater) during COVID-19-related restrictions (Gotthardt et al., 2022). Internet-based digital cultural consumption has seen its main consumers shifting from offline to online, which has become an important factor in driving economic recovery. Some scholars have pointed out that cultural and creative products should focus on consumers’ inner feelings and spiritual experiences (Gao et al., 2018; Radermecker, 2021). Therefore, cognitive engineering has been widely used in the sectors of creative design in recent years. And cognitive engineering has been combined with information dissemination theory to help people deeply understand the communication methods of artists and their audiences. All these shifts are conducive for art to be closer to our life and humanity, thus bridging the barriers and gaps caused by the creation and expanding new possibilities of cultural consumption. Digital audio and video, animation games, webcasting, etc. are all important parts of the digital cultural industry based on mobile smart terminals, mobile Internet technology, and network big data. With audio and video works as the medium, this research will study the effect of emotional design factors on the communication of audio-visual art. The specific research purposes are as follows: to explore how art creators influence the audience’s perception and dissemination through audio-visual art; to understand the key factors that audio-visual art affects the audience’s emotional experience; to deeply analyze the differences in the audience’s perception of the emotional design of musical works and film & television works.

## Literature review

### The emotional design and emotional communication mode of audio-visual art

Audio-visual artistic works can stimulate the audience’s perceptual system through certain design factors, thereby triggering emotional responses, generating emotional connections, and arousing their inner desires. People’s heartstrings are touched mainly by such works’ attributes as artistic styles, story plots, audio-visual structures, and thematic connotations (Schmetkamp, 2017). With the help of the fantasy world expressed by the screen, the audience can gain pleasant sensations intertwined with curiosity, desires, and overlapping emotions aroused by dramatic conflicts, thus obtaining some spiritual comfort (Mulvey, 1975). Bourdieu (2017) also mentioned that an artistic work would be meaningful to viewers with a cultural sensibility and arouse their interest, and people’s emotional projection would be manifested through the color, line, rhythm, style, form, or purpose of works. As coders of interpretation of artistic works, audio-visual artists are good at establishing an interdependent relationship between themselves and their works by integrating emotions into works. In this case, emotional design refers to the design in audio-visual art that can mobilize the audience’s perceptual system and generate their emotional interaction (Nicoló et al., 2022). Nowadays, with the rapid development of science and technology, design has gone beyond the realization of functional purposes, into a reflection of emotional and cultural values (Flores and Roldo, 2012). Emotional involvement is also central to the immersive experience, especially driven by auditory and visual stimuli in a virtual environment (Sangkyun, 2012; Jan et al., 2020; Salselas et al., 2021). Studies by some scholars indicated that the use of standardized visual and auditory emotional stimuli could induce different emotional states, thereby affecting the interaction process between the audience and the works, both on physiological and psychological levels (Ashby and Johnson, 2003; Zhou et al., 2013; Ding, 2021). The emotional design of art can finally penetrate the spiritual world of its audience, excavate their purest emotion, and empower them to understand the works’ deepest meaning, by which the audience can reach a higher realm of realizing the sublimation from sensory appreciation to spiritual reflection.

The audience’s perception and appreciation of audio-visual works represent the first stage of emotional communication between people and art objects. In this process, the audience is required to combine their knowledge, experience, and perception to decipher and decode (Formilan and Stark, 2021). Evoking attention by showing audio-visual artistic works activates the motor system and embodiment as the emotional–motivational state is linked with cognitive processes in human–environment interactions from a sensory-cognitive structure (Izard, 2013; Sakhaei et al., 2022). Mcluhan (2000) proposed that the medium was an extension of human beings, and audio-visual media had a strong influence on people’s touch, and thus made people’s perception a three-dimensional structure. Langer (1957) once said that any kind of art was an external manifestation of inner essence, an objective representation of subjective reality. In other words, the measurement standard of emotional design in audio-visual art includes the objective scope, and the latter’s internal structure can be reflected through correlation hierarchy. In the past few decades, many scholars have proposed evaluation criteria that affect the audience’s emotional perception. For example, Norman (2004) believed that high-quality design must take into account both aesthetics and usability, which could be evaluated through such attributes as aesthetics, attractiveness, fun, and usability. Research launched by Khalid and Helander (2004) indicated that the information value or utility, functionality and semantics, familiarity, and usability of cultural and creative products would affect the demands and preferences of the audience. These factors showed that the function of their abilities played an important role in emotional perception. Creativity comes from the yearning for a certain lifestyle, from which the symbolic meaning is extracted, transformed into a visual consumption symbol, and finally integrated into artistic works (Hsu and Lin, 2011). Works with the added value of cultural creativity, such as image code, pattern type, and modeling composition, can satisfy the emotional demands of the audience from external signs to connotation. From what has been suggested above, artists are good at integrating the three dimensions of beauty, functionality, and creativity, as well as conveying potential or implied emotions and meanings, which is also of vital importance in the construction of emotional design.

**FIGURE 1:**
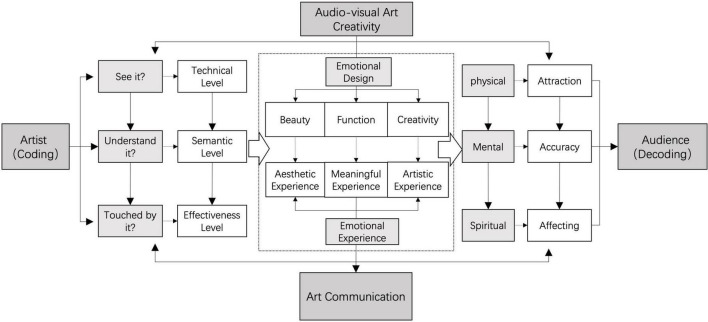
The emotional communication mode of audio-visual art.

Speaking of the emotional communication of audio-visual art, it holds creative connotation based on the emotional design of the artists and reflects the audience’s perceptive feelings with the aim of their emotional experience, both of which work to build a bridge of communication connecting artists and the audience. As the composer Cage John (2013) said, art was not the act of an artist to create well-connected works, but a place for the audience to interact with artistic works (a.k.a. art medium). In this case, artistic works become an interactive medium that the artists encode and the audience decodes. The procedural school among communication theories believes a successful encoding needs the artist to take account of three aspects, namely the technical level, the semantic level, and the effectiveness level (Jakobson, 1987; Craig, 1999; Fiske, 2010). The technical level means that the code creator sends messages that convey his intention so that artistic work can be perceived by the code recipient graphically; the semantic level means that the code sender expresses his meaning of an artistic work, which can be well understood by the code recipient authentically; the effectiveness level means that the code sender has an effective influence on the code recipient on his expectant behaviors according to the original meaning (Lin and Li, 2015; Fang et al., 2018). The audience’s emotional experience of an artistic work involves their decoding process. At the level of the body’s instinct, the audience will be attracted by the external senses of an artistic work; at the level of the mind’s behavior, they will understand and feel the meaning beyond the perception of artistic works. Eventually, they will return to the level of spiritual reflection where the audience will be touched deeply in their hearts and the artistic work will be evocative of their memories of emotion in their lives. Given that, this research combines the relevant communication and cognitive theories to give a conclusion about the emotional communication mode of audio-visual art, as shown in Figure 1.

### Emotional design evaluation matrix of audio-visual art

On the theoretical basis of the emotional communication mode of audio-visual art, this research establishes the emotional design evaluation matrix of audio-visual art to better understand the classification of evaluation attributes and provide a reference for the measurement of subsequent research structures, as shown in Table 1. The aesthetic dimension created by the artist using logical conception can condense the audience’s aesthetic experience into a profound aesthetic experience. To resonate with the audience in such three dimensions as beauty, function, and creativity, artists should make endeavors in these three aspects: technique, meaning, and effect. At the technical level, Expression techniques (A1-1) and Audio-visual language (A1-2) can be summarized as two attributes of the aesthetic dimension. Expression techniques refer to the application of techniques, such as film composition, shooting techniques, lens using, tune arrangement, and singing styles, which are the most basic design elements for expressing beauty; Audio-visual language can be generalized as an effect being represented by the internal organizational structure of an artistic work, which specifically includes the fluency of scenes switching, the richness of melody, and the vividness of the singing. Both the above attributes emphasized the technical methods that artists use to convey aesthetic feelings with the measurement reference of beauty representation. At the semantic level, the aesthetic dimension includes Rhythm and melody (B1-1) and Harmonious coexistence (B1-2). Rhythm and melody have an overlapping or interlaced effect which is similar to a sparse or dense arrangement of characters according to different meaning rules. Given that, the sense of beauty is more like insight beyond agreeable sensations; Harmonious coexistence means that all elements integrate subtly as one to create an aura of graceful harmony. So these two attributes can reflect the inside beauty of the artwork through the subtle organization with the measurement reference of beauty enjoyment. At the effectiveness level, Artistic charm (C1-1) and Aesthetic value (C1-2) can be extracted from the aesthetic dimension. Artistic charm actually reshapes the relationship between artists and their works, both of which exist because of art, the original third party that gives people a unique perceptive feeling of artistic events (Heidegger, 2011); Aesthetic value contains imagination, expression, emotion, motivation, transformation and many ways to realize the beauty of an artistic work, and it is a reproduction of beauty of the aesthetic creation (Langer, 1957). In general, these two attributes are the objective expression of the audience’s inner essence of aesthetic experience.

**TABLE 1:** Emotional design evaluation matrix of audio-visual art.

Artist				
Code\Measurement indicators	Beauty	Function	Creativity	
Technical Level (See?)	A1-1 Expression techniques A1-2 Audio-visual language	A2-1 Artistic style A2-2 Thematic perception	A3-1 Content conception A3-2 Scene creation	Physical (Attraction)
Semantic Level (Understand?)	B1-1 Rhythm and melody B1-2 Harmonious coexistence	B2-1 National culture B2-2 Spirit of times	B3-1 Emotion presupposition B3-2 Imagination stimulation	Mental (Accuracy)
Effectiveness Level (Touched?)	C1-1 Artistic charm C1-2 Aesthetic value	C2-1 Implication and connotation C2-2 Thought promoting	C3-1 Emotional resonance C3-2 Spiritual sublimation	Spiritual (Affecting)
	**Aesthetic experience**	**Meaningful experience**	**Artistic conception**	**Emotional experience\Decode**

**Audience**				


The functional value of audio-visual artistic works is to create emotions through pure forms of expression, such as virtual images or musical notes, to draw forth the audience’s certain sentiments and make them taste the deepest connotation of artistic works. Therefore, in terms of the functional dimension, different attributes are based on such levels as technique, meaning, and effect. At the technical level, the functional dimension can be divided into Artistic style (A2-1) and Thematic perception (A2-2). Artistic style is an exceptionally expressive way of indicating the interconnection of space, time, and events in the audio-visual world; Thematic perception means that an artistic work profoundly expresses its purpose and meaning through external forms and thus reveals its theme. As Goodman (1990) said in Languages of Art, creating an artistic work was a process of building its structure where existed the abstraction and expression of its style, the establishment and modification of its purposes, and the distinction and connection of its thematic elaborating methods. At the semantic level, two main attributes of the functional dimension are National culture (B2-1) and Spirit of times (B2-2). The National culture in a video embodies national feelings and cultural deposits, basically, with the use of symbolism, regional customs, linguistic structure, etc.; the social environment where art attaches and maintains its status empowers artistic works with the Spirit of times, which can be described as a core value philosophy under specific circumstances. These two attributes provide the audience with a deep interpretation of audio-visual artistic works at the functional level. At the effectiveness level, two main features are Implication and connotation (C2-1) and Thought promoting (C2-2). Art is a reflection of people’s desire for knowledge, and researches on its production, nature, and audience are of better help for people to understand its potential order and value (Grosse, 1987). Therefore, on the one hand, Implication and connotation are likely to be a unity of artistic works’ artistry and ideology, and an implication of possibilities of being understood deeply; On the other hand, an artistic work will lead people to think based on recognizing its meaning, which is called Thought promoting effect. Just as a literary critic Bryson (1983) put forward, art was a composition of various cultural symbols whose symbiosis relationship between connotation and denotation was highlighted by art in turn. Art tends to represent universal truths about things, not only in the way as it was and as it is thought to be, but also as it ought to be (Aristotle, 1989). So the functional dimension of audio-visual artistic works is to realize the expected effect of the works’ meaning as they ought to be, based on the technical and meaning-express levels, as well as to deliver the true content of art.

The essence of art is artists’ sentimental illusion, a reaction to some ideas, and the audience will obtain artistic conceptions beyond the outside from the independent existence of this kind of illusion and the space with certain forms (Langer, 1957). At the technical level of the creative dimension, the two attributes of Content conception (A3-1) and Scene creation (A3-2) are summarized. Content conception means that the ingenious structure of artistic works makes people feel touched and then internalizes this feeling into an almighty tension. In other words, the diversity, complexity, and infinity inside artistic works will create an invisible force to be attractive and intriguing; Scene creation refers to the unique scenes and atmosphere of artistic works where the audience can be personally on the scene and immerse themselves in the emotions. Given that, both two attributes are objective conditions for creative performances of audio-visual artistic works. In the semantic level of the creative dimension, the two attributes are Emotion presupposition (B3-1) and Imagination stimulation (B3-2). To realize the design of artistic conception conforming to normal logic, Emotion presupposition will be used to combine the creative rules of artistic works with their texts; Imagination stimulation becomes the key to developing the audience’s spiritual feeling. Under the narrative structure, the audience will have associations with individuals, society, and culture and imagine an infinite space with the symbols of music and scenes. These two attributes are semantic-level strategies artists use to give play to their creativity. In the effectiveness level of the creative dimension, there are also two attributes, which are Emotional resonance (C3-1) and Spiritual sublimation (C3-2). To imagine an artistic work is to imagine the way of life it works. Only by reconstructing the relevant system of meaning like an artist can people grasp the emotions conveyed by art (Danto, 2007). That is why Emotional resonance becomes an inner purpose of realizing aesthetic creativity; At the same time, what becomes the result is Spiritual sublimation, which arouses the audience’s latent desires after their perception of art. As Nietzsche (1872) stated in The Birth of Tragedy, that the art could save people could not be put down to its moral connotations but could be put down to the Dionysian spirit. People would have an eternal spiritual bailment after the Dionysian spirit making them realize the pain and cruelty of life. It can be seen from this that the essence of creative audio-visual art is the emotional comfort and the purification of the soul through aesthetic forms.

### The dialectical relationship between audio-visual emotional design, emotional experience, and sharing intention

#### Audio-visual emotional design and emotional experience

Audio-visual art needs to take the audience’s emotional feelings as the priority. Empathy is one of the design factors that artists take into account and only artistic works with the ability to reflect this kind of empathy can remind the audience of their life experience, thus realizing a sense of identity and empathy. Arnheim (2003) proposed that people’s psychology, including feelings, perception, and memory, could be conceptualized as a hierarchical feedback network by which everyone could have the same reference from a different branch of the network. In other words, the emotional design provides a basis for this reference, establishing emotional tone with abstract audio-visual language, and then constructing a psychological situation from various dimensions to achieve the goal of emotional description. As a psychological phenomenon, the emotional experience usually includes joy, passion, movement, excitement, happiness, etc. An event (i.e., a stimulus) triggers one of several stereotyped responses in the brain and body (Wilson-Mendenhall et al., 2013). The emotions are experienced primarily as structures of feeling which give meaning to relational experience (Burkitt, 2014). Given that, a high-quality design, as a stimulus, must be able to arouse the audience’s inner feelings and a certain emotion, so the emotional design is highly related to emotional experience.

#### Audio-visual emotional design and sharing intention

From the perspective of sociology, Arnold (1987) believed that art could not realize its value without sharing functions, or it could only be defined as some simple words, symbols, or pictures. True art is a form of communication more than expression. As far as the semantic school of communication theory is concerned, an artistic work successfully expressed through a symbolic system must embrace three functions: signification, impression, and communication (Barthes, 1967; Jakobson, 1987). Art can satisfy people’s spiritual needs. To fully convey emotional expressions, art language needs to convey emotional meaning through an independent structure based on the functions of signification and impression. As a result, the communication function will be achieved by experience sharing between artistic works and the audience. In this process, how emotions are organized in art becomes the key to satisfying the audience’s emotional needs and is also an important factor for information dissemination. Given that, the motivation of audio-visual emotional design is based on the audience’s demands, and the main approach of its spread is the audience’s sharing intention. Besides, artists’ creation of emotional design aims at great communication.

#### Emotional experience and sharing intention

The audience, or consumers of audio-visual artistic works, can be seen that their inner feelings and potential needs have a positive influence on their actions and decisions. In the process of enjoyment of artistic works, they play an active role, which is that they gain an experience of interaction when they have a perception during the conversation with artistic works. This process indicates that the audience participates in the creation and subsequent sharing of emotional experience they obtain from artistic works is exactly the behavioral result. This is so in everyday life where perception, emotion, action, cognition, and the world tend to fuse (Crippen, 2021). Fictional emotions evoked by art can motivate actions of various kinds (Werner, 2020). As art is complete only after being accepted by people, the connection between the audience and audio-visual artistic works illustrates the completion of appreciation activities. Barthes (2002) stated that the birth of readers must be at the expense of the author’s death. Once the audience obtained the ability of independent thinking about art, they could fill in the blanks of the artistic works themselves, only leaving an opportunity for the production of some kind of relationship between artistic works and the audience. The study finds that emotional experience has a significant and positive effect experience symbolic consumption (Tangsupwattana and Liu, 2018). A better understanding of the interrelations between the emotional experience and the sharing intention can become an important reference for the audience’s art consumption. Meanwhile, artists can activate the audience’s unconscious and in-depth emotions by using the orientation, methods, and strategies of creation and can influence communication behavior and life orientation.

## Methodology

### Research structures and presuppositions

**FIGURE 2:**
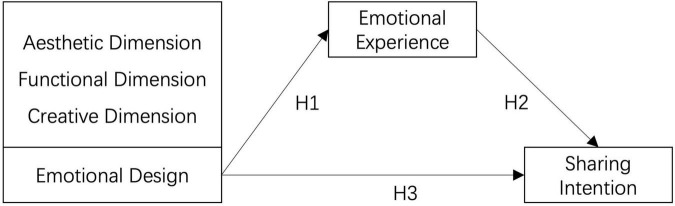
Research structure.

This research explores the audience’s emotional experience and sharing intention of audio-visual artistic works from the perspective of emotional design. The emotional design consists of three measurement dimensions, namely the aesthetic dimension, the functional dimension, and the creative dimension. The structure of this research is also designed with the use of relevant theories, document analysis (Figure 2), and emotional experience as an intermediate variable. The purpose of this design is to discuss whether emotional design can affect the audience’s sharing intention. According to the NO.1 research purpose listed in chapter one, the research come up with the following presupposition: Ha1 (Emotional design has a significant influence on emotional experience in musical works); Ha2 (Emotional experience has a significant influence on sharing intention in musical works); Ha3 (Emotional design has a significant influence on sharing intention in musical works); Ha-1 (Emotional experience has an intermediate effect on emotional design and sharing intention in musical works); Ha-2 (Emotional design has an impact on emotional experience and then promote people’s sharing intention in musical works); Hb1 (Emotional design has a significant influence on emotional experience in film & television works); Hb2 (Emotional experience has a significant influence on sharing intention in film & television works); Hb3 (Emotional design has a significant influence on sharing intention in film & television works); Hb-1 (Emotional experience has an intermediate effect on emotional design and sharing intention in film & television works); Hb-2 (Emotional design has an impact on emotional experience and then promote people’s sharing intention in film & television works). According to NO.2 research purpose, the research comes up with the following presupposition: there are different key factors affecting the emotional experience of musical works as well as film & television works. According to NO.3 research purpose, the research comes up with the following presupposition: Significant differences appear in the audience’s evaluation of music, films, and television in the level of emotional design.

### Research objects

In order to verify the evaluation matrix and the research structure of emotional experience in audio-visual artistic works, researchers make evaluations with two original works created by themselves as objects, which are an anti-epidemic song and a short film *China Braves Headwind*. The details of the lyrics are shown in Table 2.

**TABLE 2:** The music *China Braves Headwind.*

	Mom says always remember	This year’s extraordinary winter	People of all ethnic groups	We brave headwinds together
Lyric	Don’t need to be scared of troubles	In the way of the national revival	As long as we stick as one	Success will come to all
	Medical workers are already in the field	They are Nightingales of the new era	We ride the waves freely with no fear	Under the shelters of their invincible will

### Questionnaire design

The questionnaire is divided into two parts. The first part is the basic information of the subjects, including gender, age, academic degree, and educational background; The second part lists 22 questions on the evaluation matrix of emotional experience in the audio-visual artistic works, using the Likert scale (1 represents the lowest degree of agreement and 5 represents the highest degree of agreement). With the Likert scale, subjects are asked to rate the two objects, the music, and the short film both named *China Braves Headwind*. Questions 1 to 6 cover categories of aesthetics, which are based on six factors: expression techniques, audio-visual language, rhythm and melody, harmonious coexistence, artistic charm, and aesthetic value; Questions 7 to 12 cover categories of function, which are based on six factors: artistic style, thematic perception, national culture, the spirit of times, implication and connotation, and thought promoting; Question 13 to 18 cover categories of creativity, which are based on six factors: content conception, Scene creation, emotion presupposition, imagination stimulation, emotional resonance, and spiritual sublimation. Questions 19 to 21 cover categories of emotional experience which are based on three factors: preference degree, moving degree, and inspiring degree; Question 22 covers the category of sharing intention which is based on the extent people are likely to share artistic works with others. Questionnaire star platform is used to produce such questions and the websites are https://www.wjx.cn/jq/65312477.aspx and https://www.wjx.cn/jq/65057119.aspx.

### Experimental design

The research samples are teachers and students from various colleges and universities, ranging in age from 19 to 40 years. Among them, subjects participating in the questionnaire about music include 90 men and 284 women, and subjects participating in the questionnaire about films include 73 men and 253 women, who have undergraduate and postgraduate degrees and have various learning backgrounds, such as art, science and engineering, humanities, and social science, as is shown in Table 3. This research conforms to ethical requirements and respects the privacy of the subjects. There is no sex discrimination, racial discrimination, and other issues. The questionnaires are mainly distributed online where researchers ask for the subjects’ agreement to fulfill the questionnaires. Researchers convene the subjects through Tencent Conference software as an online laboratory. At first, researchers elaborate on their purpose and the instructions for answering the questions in detail before playing the music *China Braves Headwind*. After the play of music, subjects are allowed to fulfill the first questionnaire in 10 min. Then, they have 10 min to fulfill the second questionnaire after watching the short film *China Braves Headwind*. After the questionnaires are submitted, researchers discard samples that cannot meet the conditions after reviewing them. The standard is that questionnaires with the same scores for too many questions are eliminated. Finally, 374 valid questionnaires for the music test and 326 for the film test are collected, with subjects 17 times more than the data. In the Structural Equation Modeling analysis, some scholars’ research show that the demand for the number of samples is related to the length of the research scale, and the longer the research scale is, the more samples will be required. The ideal number of samples is at least five times more than the number of measurement variables, for which the number of samples should be between 200 and 400 (Ghiselli et al., 1981; Gorsuch, 1983; Hair et al., 1998; Wu and Tu, 2010). Therefore, the samples taken in this research meet the needs of the number of samples for research and analysis.

**TABLE 3:** Profile of the respondents.

Characteristics	Levels	Film and television works	Musical works
		Number	Number
Gender	Male	73	90
	Female	253	284
Age	19–30	315	363
	31–40	11	11
Education	Undergraduate	306	350
	Postgraduate	20	24
Academic degree	Arts	62	78
	Science and engineering	93	121
	Humanities and social science	171	175

The questionnaire was subject to reliability and validity tests, regression analysis and dependent samples *t*-test with statistical tests using SPSS 22.0, as well as structural equation modeling (SEM) in AMOS 22.0. Whether the measurement scale is feasible can be confirmed by verification factors. And then through the path diagram of SEM, the presuppositions can be verified followed by multiple regressive analyses to explore the key factors affecting the audience’s emotional experience. Finally, the research will study the audience’s differences in emotional design in the music and the short film, using the dependent samples *t*-test.

## Results

### The analysis of structural model construction

#### Convergent validity and discriminant validity

At this stage, the research uses the method of measurement model analysis. Based on valid questionnaires on the music *China Braves Headwind*, the research launches the model construction analysis and deals with the structural relationship between the observed variables and latent variables through confirmatory factor analysis (CFA). And then, convergent validity and discriminant validity in the measurement model are tested. According to the findings through CFA, in terms of factor loadings, the beauty dimension ranges from 0.772 to 0.844; the function dimension from 0.683 to 0.843; the creativity from 0.735 to 0.866; emotional experience dimension from 0.831 to 0.922; emotional design dimension from 0.910 to 0.978. All factor loadings are more than 0.5, showing that the measurement model meets the standard, as shown in Table 4. What’s more, the composite reliability (CR) of all dimensions in the research ranges between 0.897 and 0.956, and the average variance extracted (AVE) varies between 0.612 and 0.879. Both of them conform to the values 0.60 and 0.50 — advised ones in related research (Fornell and Larcker, 1981; Bagozzi and Yi, 1988; Hair et al., 1998). The data indicate the internal consistency in the model is acceptable and has convergent validity. When it comes to the verification analysis of discriminant validity, The value of the square root of AVE for every dimension in the research is 75% more than the proportion of the total number of correlation coefficients for every dimension of the total compared number. It shows there is desirable discriminant validity among the variables (Hair et al., 1998).

**TABLE 4:** The research model confirmatory factor analysis table.

Dimension	Items	Skewness	Kurtosis	Parameter saliency estimation				Factor loadings	Question reliability	Composite reliability	Convergent validity
								
		SK	KU	Unstd.	S.E.	t-value	P	Std.	SMC	CR	AVE
Beauty	A1-1	−0.502	0.081	1.000				0.800	0.640	0.919	0.653
	A1-2	−0.527	–0.213	0.996	0.058	17.095	***	0.798	0.637		
	B1-1	−0.685	0.007	1.056	0.060	17.538	***	0.814	0.663		
	B1-2	−0.486	–0.420	1.045	0.059	17.704	***	0.820	0.672		
	C1-1	−0.402	–0.426	1.193	0.065	18.397	***	0.844	0.712		
	C1-2	−0.348	–0.387	1.020	0.062	16.366	***	0.772	0.596		
Function	A2-1	-0.737	0.087	1.000				0.683	0.466	0.904	0.612
	A2-2	−1.121	1.011	0.979	0.075	13.105	***	0.747	0.558		
	B2-1	−0.942	0.480	0.949	0.071	13.318	***	0.761	0.579		
	B2-2	−0.886	0.357	1.024	0.071	14.488	***	0.838	0.702		
	C2-1	−0.760	0.150	1.151	0.079	14.553	***	0.843	0.711		
	C2-2	−0.941	0.898	1.012	0.072	14.049	***	0.808	0.653		
Creativity	A3-1	−0.628	0.101	1.000				0.735	0.540	0.925	0.675
	A3-2	−0.499	–0.485	1.245	0.074	16.874	***	0.866	0.750		
	B3-1	−0.615	–0.242	1.176	0.072	16.316	***	0.839	0.704		
	B3-2	−0.321	–0.668	1.252	0.079	15.856	***	0.817	0.667		
	C3-1	−0.863	0.342	1.208	0.073	16.486	***	0.847	0.717		
	C3-2	−0.679	0.079	1.115	0.070	15.902	***	0.819	0.671		
Emotional experience	D	−0.367	–0.377	1.000				0.831	0.691	0.897	0.745
	F	−0.458	–0.237	1.071	0.053	20.372	***	0.922	0.850		
	G	−0.596	0.052	0.923	0.049	18.897	***	0.833	0.694		
Emotional design	Beauty	/	/	1.000				0.924	0.854	0.956	0.879
	Function	/	/	0.896	0.062	14.548	***	0.910	0.828		
	Creativity	/	/	1.117	0.072	15.498	***	0.978	0.956		

***p < 0.001.

#### Analysis of second-order confirmatory factor analysis model fitness

**FIGURE 3:**
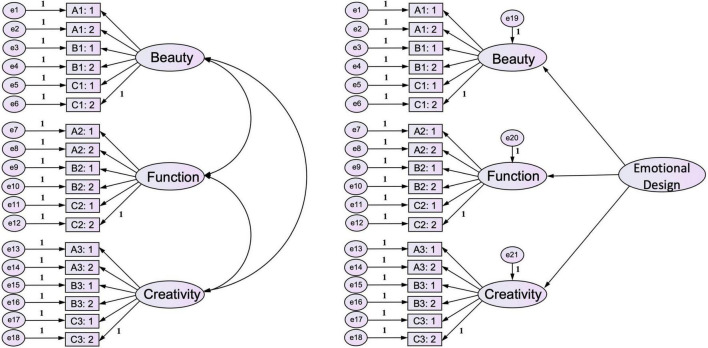
First-order three-factor model (There are correlates among these factors) and second-order factor model.

The research can decide whether to use the first-order CFA or the second-order CFA based on the concept of the targeted factor (Marsh and Hocevar, 1985). According to the fitness related to the first-order model, this target factor is removed by the fitness of the second-order model. If the result is closer to 1, that indicates that the second-order model is more streamlined, and the second-order model can represent the first-order model. In the research, the research designs and compares the first-order three-factor model (there are correlates among these factors) and the second-order factor model (Figure 3). The result shows χ^2^(Model1)/χ^2^(Model2) = 1 and the target factor of the model is 1 (Table 5). Therefore, the research uses the result of the second-order CFA to analyze the model construction (Lai et al., 2010). The study indicates that there are correlates among three factors, such as beauty, function, and creativity. They are key factors in emotional design. It is reasonable to use the second-order CFA in the research.

**TABLE 5:** Second-order model confirmatory factor analysis table.

Second-order CFA	χ^2^ value	Degree of freedom (df)	χ^2^/df	GFI	AGFI	CFI	RMSEA
1. Model 1: First-order Three-factor Model (There are correlates among these factors)	397.503	132.000	3.011	0.896	0.865	0.951	0.073
2. Model 2: Second-order Factor Model	397.503	132.000	3.011	0.896	0.865	0.951	0.073
The Advised Value	The smaller, the better	The larger, the better	<5	>0.8	>0.8	>0.9	<0.08

#### The overall model fitness test

The structural equation model (SEM) assumed in this research tests its multivariate normality in two aspects. One is the normality of observed variables, and the other is the multivariate normality after observed variables integration. Some scholars put forward that when the absolute values of the skew coefficient and kurtosis coefficient of observed variables are less than 2, it can be determined that it has normality (Bollen and Long, 1993; Qiu, 2006; Yen, 2018). From Table 4, the research can figure out that the absolute values of the skew coefficient and kurtosis coefficient of all observed variables in the structural equation model in the research are less than 2. Therefore, the research can safely conclude that the observed variables have normality. What’s more, the tested multivariate value is 42.448 > 5, which does not meet the multivariate normality standard (Barbara, 2016). When the data of SEM does not have multivariate normality, Bollen–Stine bootstrap can be used to correct standard errors and the statistics of fitness (Bollen and Stine, 1992; Enders, 2005; Fisher and King, 2010). After Chi-square correction, the research tests the model fitness. The results show that the ratio of chi-square value to degree of freedom range from 1 to 3 (χ^2^/DF = 1.35), GFI = 0.962 > 0.9, AGFI = 0.95 > 0.9, RMSEA = 0.031 < 0.08, RMR = 0.041 < 0.08, TLI(NNFI) = 0.989 > 0.9, CFI = 0.99 > 0.9, IFI = 0.99 > 0.9, and Hoelter’s N(CN) = 277.528 > 200. From what has been suggested above, the overall index of this model is acceptable (Doll et al., 1994; Baumgartner and Homburg, 1996; Hair et al., 2010; Chen and Wang, 2011). In the study, the theoretical structure of the overall structural model is well-matched with the empirical data and the model has desirable construct validity.

### Model research hypothesis testing

**FIGURE 4:**
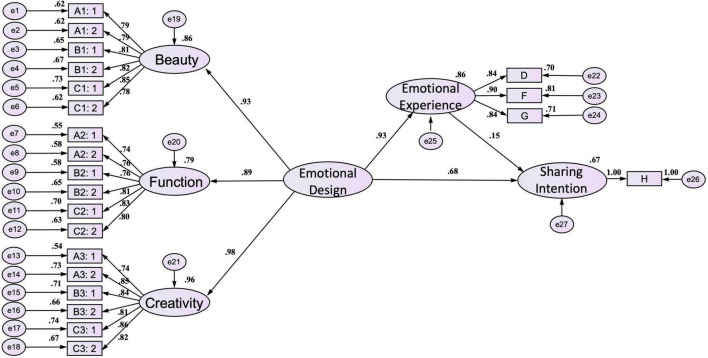
Path diagram of musical works structure model in the research.

**FIGURE 5:**
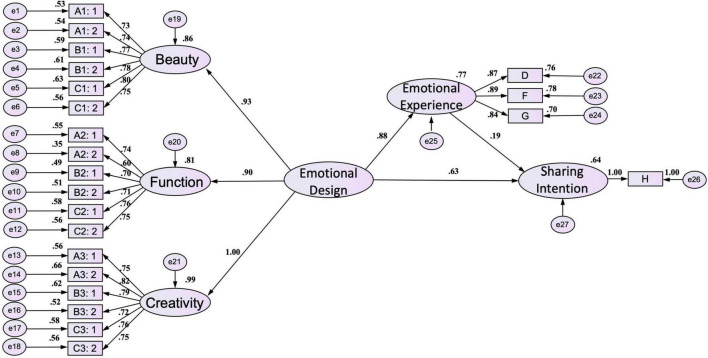
Path diagram of film & television works structure model in the research.

In the research, the SEM is used to test the validation of research hypotheses. After the research and analysis, there are six dimensions and 22 measurement variables. Results of the path diagram of musical works structure model analysis are shown in Figure 4 and Table 6 — Ha1: emotional design in musical works has a notable influence on emotional experience; Ha2: emotional experience in musical works has a notable influence on sharing intention; Ha3: emotional design in musical works has a notable influence on sharing intention. Therefore, the above three hypotheses are true. Results of the path diagram of film & television works structure model analysis are shown in Figure 5 and Table 6 — Hb1: emotional design in film & television works has a notable influence on emotional experience; Hb2: emotional experience in film & television works has a notable influence on sharing intention; Hb3: emotional design in film & television works has a notable influence on sharing intention. Therefore, the above three hypotheses are true.

**TABLE 6:** Path coefficient of structural statistical model.

Type	Path	Coefficient (Variant)			Standardized path coefficient	t-value C.R.	P	Decision
Musical works	Ha1	Emotional Design	->	Emotional Experience	0.930	17.384	***	Supported
	Ha2	Emotional Experience	->	Sharing Intention	0.150	3.342	***	Supported
	Ha3	Emotional Design	->	Sharing Intention	0.679	17.384	***	Supported
Film & Television works	Hb1	Emotional Design	->	Emotional Experience	0.875	14.299	***	Supported
	Hb2	Emotional Experience	->	Sharing Intention	0.187	3.897	***	Supported
	Hb3	Emotional Design	->	Sharing Intention	0.630	14.299	***	Supported

***p < 0.001.

The research has shown that emotional design, emotional experience, and sharing the intention of audio-visual works are influenced by each other. The research can conclude that the hypotheses are true. Ha-1: in the musical works, emotional experience plays a mediating role between emotional design and sharing intention; Ha-2: in the musical works, the emotional design will impact emotional experience, further improving sharing intention. Hb-1: in the film & television works, emotional experience acts as an intermediary between emotional design and sharing intention; Hb-2: in the film & television works, the emotional design will impact emotional experience, further improving sharing intention.

To test the indirect effect of emotional design on sharing intention and the mediating effect of emotional experience in musical works, the research uses the Sobel-Goodman tests. The results show that the Z value is 3.264, more than the standard value of 1.96, indicating the mediating effect is remarkable (Sobel, 1982, 1986). And then, The research uses the Bootstrap techniques for error estimation to continue the hypotheses test and re-estimate the confidence level, standard error, standardized coefficient, and significant level (Z value) of indirect effect (MacKinnon et al., 2007; MacKinnon, 2008; Taylor et al., 2008). The 95% confidence interval of the indirect effect path of emotional design on sharing intention does not contain zero. Its Bias-corrected (0.115, 0.337), percentile (0.110, 0.333), and *p* < 0.001 indicate the significance and the direct effect is not zero; the Z value of the indirect effect is 3.911, more than 1.96 and the results show the significance and there is the mediating effect, as shown in Table 7. In addition, the Z value of the direct effect is 16.400, more than 1.96, and the results show significance, indicating that the emotional design does have a direct effect on sharing intention (Table 7). Therefore, all results show there is a mediating effect and it plays the role of partial mediation, and they can prove the hypothesis Ha-1: in the musical works, emotional experience plays a mediating role between emotional design and sharing intention. From the standardized path coefficient in the model, the research can know that the influence path coefficient of emotional design on sharing intention (direct effect) is 0.68; the path coefficient of emotional design on sharing intention (indirect effect) is 0.140 (0.93*0.15); the total effects are 0.93*0.15 + 0.68 = 0.82 > 0.68 (total effects > direct effect). All data can prove hypothesis Ha-2: in the musical works, the emotional design will impact emotional experience, further improving sharing intention.

**TABLE 7:** The standardized indirect, direct, and total mediating effects in the musical works structural model.

Variants	Point estimation value	Product of coefficients		Bias-corrected 95% CI		Percentile 95% CI		Two-tailed significance
		SE	Z	Lower	Upper	Lower	Upper	
**Musical works**								
**Standardized total effects**								
Emotional design-> Sharing intention	1.285	0.067	19.179	1.169	1.428	1.165	1.426	0.000***
**Standardized indirect effect**								
Emotional design-> Sharing intention	0.219	0.056	3.911	0.115	0.337	0.110	0.333	0.000***
**Standardized direct effect**								
Emotional design-> Sharing intention	1.066	0.065	16.400	0.948	1.201	0.950	1.202	0.000***
**Film and television works**								
**Standardized total effects**								
Emotional design-> Sharing intention	1.302	0.090	14.467	1.131	1.502	1.130	1.501	0.000***
**Standardized indirect effect**								
Emotional design-> Sharing intention	0.268	0.083	3.229	0.102	0.421	0.100	0.420	0.000***
Standardized direct effect								
Emotional design-> Sharing intention	1.034	0.085	12.165	0.869	1.201	0.878	1.211	0.000***

***p < 0.001.

To test the indirect effect of emotional design on sharing intention and the mediating effect of emotional experience in film & television works, the research uses the Sobel-Goodman tests. The results show that the Z value is 3.7332, more than the standard value of 1.96, indicating the mediating effect is remarkable. And then, The research uses Bootstrap techniques for error estimation to continue the hypotheses test and re-estimate the confidence level, standard error, standardized coefficient, and significant level (Z value) of indirect effect. The 95% confidence interval of the indirect effect path of emotional design on sharing intention does not contain zero. Its Bias-corrected (0.102, 0.421), percentile (0.100, 0.420), and p < 0.001 indicate the significance and the direct effect is not zero; the Z value of the indirect effect is 3.229, more than 1.96 and the results show the significance and there is the mediating effect, as shown in Table 7. In addition, the Z value of the direct effect is 12.165, more than 1.96, and the results show significance, indicating that the emotional design does have a direct effect on sharing intention. Therefore, all results show there is a mediating effect and it plays the role of partial mediation, and they can prove the hypothesis Hb-1: in the film & television works, emotional experience plays a mediating role between emotional design and sharing intention. From the standardized path coefficient in the model, the research can know that the influence path coefficient of emotional design on sharing intention (direct effect) is 0.63; the path coefficient of emotional design on sharing intention (indirect effect) is 0.167 (0.88*0.19); the total effects are 0.88*0.19 + 0.63 = 0.797 > 0.63 (total effects > direct effect). All data can prove hypothesis Hb-2: in the film & television works, the emotional design will impact emotional experience, further improving sharing intention.

### The analysis of key factors affecting emotional experience with mediating effect

All the above research has proven that the emotional design both in musical works and film & television works has a significant effect on the audience’s emotional experience. And, emotional experience with mediating effect serves as the emotional bond between the artists’ creation and the audience’s perceptions and their sharing intention. At this stage, the research will take the audience’s emotional experience as a dependent variable and nine evaluation factors listed in the Emotional Design Evaluation Matrix of Audio-visual Art as indicators and predictive variables of emotional design in the works. Through these efforts, the research will carry on the multivariate regression analysis on the factors affecting the audience’s emotional experience and further explore the impacts of nine factors on emotional experience.

As is shown in Table 8, from all predictive variables and the correlation between the audience and the intensity of emotional experience in the musical works, the research can know nine predictive variables and their correlation coefficients are 0.703, 0.740, 0.737, 0.720, 0.720, 0.602, 0.650, 0.721, 0.766, 0.820, meeting the significant level 0.001. From the multivariate regression analysis in Table 8, the research can find that the correlation coefficient R between overall prediction variables and dependent variables is 0.869. And the explanatory variance of nine predictive variables on the intensity of the emotional experience is 75.6% and the F value is 73.291, meeting the significant level, 0.000. According to the results, there are significant correlations between the nine attributes and the intensity of emotional experience, and these nine attributes have a considerable degree of joint explanatory power for the intensity of the emotional experience. Among these attributes, the more prominent ones in order are A1 (Expression techniques, Audio-visual language), B1 (Rhythm and melody, Harmonious coexistence), A2 (Artistic style, Thematic perception), B2 (National culture, Spirit of times), B3 (Emotion presupposition, Imagination stimulation), and C3 (Emotional resonance, Spiritual sublimation). All these attributes represent key factors affecting the intensity of emotional experience in musical works.

**TABLE 8:** The regression analysis of emotional experience.

Type	Dependent variables	Predictive variables	Simple correlation	Regression coefficient	Standardized	T value	Significance level
					regression		
					coefficient		
Musical works	The Intensity of “Emotional Experience” (*N* = 374)	A1	0.703***	0.117	0.106	2.432*	0.015
		B1	0.740***	0.159	0.147	3.015**	0.003
		C1	0.737***	0.063	0.062	1.236	0.217
		A2	0.720***	0.130	0.116	2.577*	0.010
		B2	0.602***	–0.116	–0.093	−2.013*	0.045
		C2	0.650***	0.049	0.042	0.899	0.369
		A3	0.721***	0.053	0.050	1.003	0.316
		B3	0.766***	0.128	0.129	2.548*	0.011
		C3	0.820***	0.425	0.410	8.240***	0.000
		Invariable –0.237					
		R = 0.869 Rsq = 0.756 F = 125.175 SigmfF = 0.000					
Film and Television works	The Intensity of “Emotional Experience” (*N* = 326)	A1	0.613***	0.073	0.068	1.341	0.181
		B1	0.687***	0.113	0.108	1.899	0.058
		C1	0.643***	0.016	0.017	0.312	0.755
		A2	0.664***	0.153	0.119	2.253*	0.025
		B2	0.522***	–0.071	–0.056	–1.169	0.243
		C2	0.608***	0.066	0.060	1.141	0.255
		A3	0.721***	0.208	0.199	3.308**	0.001
		B3	0.711***	0.114	0.110	1.837	0.067
		C3	0.743***	0.350	0.325	6.102***	0.000
		Invariable –0.220					
		*R* = 0.822 Rsq = 0.676 *F* = 73.291 SigmfF = 0.000					

*p < 0.05, **p < 0.01, ***p < 0.001.

From all predictive variables and the correlation between the audience and the intensity of emotional experience in the film & television works, the research can know nine predictive variables and their correlation coefficients are 0.613, 0.687, 0.643, 0.664, 0.522, 0.608, 0.721, 0.711, 0.743, meeting the significant level, 0.001. From the multivariate regression analysis in Table 8, the research can find that the correlation coefficient R between overall prediction variables and dependent variables is 0.822. And the explanatory variance of nine predictive variables on the intensity of “emotional experience” is 67.6% and the F value is 73.291, meeting the significant level, 0.000. According to the results, there are significant correlations between the nine attributes and the intensity of emotional experience, and these nine attributes have a considerable degree of joint explanatory power for the intensity of the emotional experience. Among these attributes, the more prominent ones in order are A2 (Artistic style, Thematic perception), A3 (Content conception, Scene creation), and C3 (Emotional resonance, Spiritual sublimation). All these attributes represent key factors affecting the intensity of emotional experience in film & television works.

### Analysis of difference in perception of emotional design

At this stage, to explore the audience’s perception of evaluation attributes in different types of works with the same theme, the research employed the dependent sample *t*-test to test the evaluation factors of musical works and film & television works. The analysis is shown in Table 9. Among nine evaluation factors, significant differences between musical works and film & television works are seen in attribute C1, attribute C2, affection level, touch level, inspiration level, and sharing intention. The comparative analysis of the average score of these evaluation factors shows that the average score of the film & television works is higher than that of the musical works. Specifically, there is a notable difference in the two types in attribute A1 and attribute B1 (*P* < 0.05), with the significance level of 0.012 and 0.028, respectively; there is a notable difference in the two types in attribute B2, attribute A3 and attribute B3 (*P* < 0.01), with the significance level of 0.003, 0.005, and 0.006; there is a well-significant difference in in the two types in attribute A2, attribute C3, element D, element F, element G, and element H (*P* < 0.001), with the significance level of 0.000.

**TABLE 9:** The table of analysis of differences in testers’ perception of emotional design.

Items	Type	Number	M	SD	t value	Significance level	Difference comparison
A1	Music F & T	374 326	3.930 4.075	0.7890 0.7123	−2.531*	0.012	Music < F & T
B1	Music F & T	374 326	4.003 4.132	0.8080 0.7345	−2.202*	0.028	Music < F & T
C1	Music F & T	374 326	3.822 3.914	0.8585 0.7925	–1.464	0.144	Music < F & T
A2	Music F & T	374 326	4.186 4.416	0.7703 0.5947	−4.446***	0.000	Music < F & T
B2	Music F & T	374 326	4.326 4.474	0.6951 0.5962	−3.027**	0.003	Music < F & T
C2	Music F & T	374 326	4.218 4.271	0.7476 0.6911	–0.979	0.328	Music < F & T
A3	Music F & T	374 326	3.957 4.124	0.8265 0.7279	−2.818**	0.005	Music < F & T
B3	Music F & T	374 326	3.890 4.058	0.8779 0.7388	−2.747**	0.006	Music < F & T
C3	Music F & T	374 326	4.068 4.288	0.8389 0.7101	−3.718***	0.000	Music < F & T
D: Affection level	Music F & T	374 326	3.626 3.975	0.9927 0.8482	−5.027***	0.000	Music < F & T
F: Touch level	Music F & T	374 326	3.786 4.058	0.9588 0.8663	−3.945***	0.000	Music < F & T
G: Inspiration level	Music F & T	374 326	3.939 4.169	0.9140 0.7878	−3.579***	0.000	Music < F & T
H: Sharing intention	Music F & T	374 326	3.703 3.975	1.0837 0.9279	−3.581***	0.000	Music < F & T

F & T refers to the film & television works, *p < 0.05, **p < 0.01, ***p < 0.001.

## Discussion

The model construction analysis in the research shows that the construct validity is desirable, the scales of the research are feasible, and the Emotional Design Evaluation Matrix of Audio-visual Art is reasonable. The research explained the emotional design and demonstrated the way of coding by artists from three dimensions, such as beauty, function, and creativity. There are nine evaluation factors in three dimensions, measuring the effects and experience of these dimensions from the levels of techniques, semantics, and effects. Through these efforts, the research has found that the emotional design will attract the audience, help them perceive the creation, and touch them.

According to the structural equation model survey, the emotional design of musical and film & television works will have an impact on the audience’s sharing intention. Specifically, the emotional design has a significant positive effect on the emotional experience; the emotional experience has a significant positive effect on the sharing intention; the emotional design has a significant positive effect on the sharing intention; the emotional experience serves as a partial mediator between emotional design and sharing intention. The emotional design of both musical and film & television works has a significant effect on the audience’s emotional experience, further improving their sharing intention. It has been suggested digital cultural offerings increased optimism indirectly through an increase in aesthetic experience, perceived autonomy, and relatedness (Koehler and Neubauer, 2020; Gotthardt et al., 2022). As two kinds of creative activities, musical and film & television works have their unique text processing modes. Barthes (2005) puts forward that artistic works include extension information and connotation information. The emotional design hides in connotation information through coding, which requires the audience to interpret it imaginatively. This process promotes emotion to occur. Kant (2002) believes the appreciation judgment is perceptual judgment. Through imagination, the audience will produce pleasant or unpleasant emotional connections with the subject of art. The emotional design in artistic works is the prerequisite for the audience to perceive and judge. Musical works create auditory sensual pleasure through musical notes, while film & television works stimulate emotional experience through the combination of pictures and sound. Therefore, the purpose and method of emotional design influence the audience’s experience of artistic works. Furthermore, the audience’s emotional experiences including such emotions as love, pleasure, satisfaction, excitement, and moving from the audio-visual works will positively drive their behavior and then impact how the works are communicated (Wieck et al., 2022). Sharing and communicating online during the lockdown promoted person-to-person virtual interaction which contributed to potentially social contact and cross-cultural communication (Fraser et al., 2021). After getting the emotional experience, the audience will connect works with their values and judgments so that the way of sharing and communication will be decided by the perception and feeling of the audience.

The research, through multivariate regression analysis, explores the key factors impacting the emotional experience in musical works and film & television works. The results indicate key factors affecting the emotional experience in musical works represent the level of techniques (A1, A2) and the level of semantics (B1, B2, B3) in the Emotional Design Evaluation Matrix of Audio-visual Art. And key factors affecting the emotional experience in film & television works represent the level of techniques (A2, A3) only. Therefore, attribute A2 and attribute C3 serve as common factors affecting the emotional experience in both types of art. The music *China Braves Headwind* uses exquisite artistic expression techniques in composition, arrangement, and singing, and has a rich melody, reasonable tune, vivid singing, and graceful harmony. The style of the music is infectious and the main idea of anti-epidemic conveyed is clear. From what has been suggested above, musicians must take the audience’s physical and psychological feelings into account during the COVID-19 pandemic. Only in this way, can the audience resonate with works. To present the emotional context in works, apart from the attributes in the level of techniques, creators must convey profound meanings to consumers in ways that resonate with them. During the pandemic, emotional resonance brought by music had a positive effect on the sense of happiness (Cabedo-Mas et al., 2021). As Langer (1986) said, music represents the highest level of reflection of life, that is, the symbolic expression of human emotional activities. In the short film *China Braves Headwind*, the application of various techniques like scene settings, plot arrangements, and styles produces a kind of fictional experience. They will touch the audience and make them emotional through the power of visual sense. The reason why attribute A2 (Artistic style, Thematic perception) serves as a key factor affecting the emotional experience in both musical works and film & television works is that it demonstrates works’ features from the outside to the inside. The artistic style reflects the multivariate artistic phenomenon and abstract symbols represent the connotations of works, which meets the viewers’ inner needs and further produces the empathetic effect. As for themes in works, only a clear intention can convey the creators’ purpose and significance. Therefore, only when valid information both in musical works and film & television works is conveyed, will the audience get the emotional experience. C3 (Emotional resonance, Spiritual sublimation) at the level of effects will reach deep into the soul of the audience. The attribute shows that musical works and film & television works deeply probe into the essence of life, which gives viewers spiritual support and gives birth to some emotion. In this case, the emotional experience of social belonging, empathy, and kindness have been described as critical factors for boosting social cohesion, and fostering resilience, recovery, and development from the COVID-19 pandemic (Niels et al., 2022). Therefore, this attribute serves as the most important factor in evaluating the emotional experience in audio-visual works.

Through the dependent sample *t*-test, the research has found that there is a significant difference in seven attributes, emotional experience, and sharing intention among the audience in the musical and film & television works with the same subject. And the average score of film & television works is higher than that of musical works. Therefore, compared with musical works, film & television works are likely to resonate with the audience. Although there are differences in artistic forms between the two types of art, both of them boasts endless artistic attraction and aesthetic value. The short film *China Braves Headwind* is good at combining pictures and sounds, creating a virtual-and-real situation and narrative text, enhancing the interest of the audience, and making them enter a broad space for imagination. Especially, visual representation, with which meaning is constructed through the structuring of these elements (Cano-Martínez et al., 2022), is a recreation of social, cultural, and ideological mediation (Desai, 2000; Knochel, 2013). The music *China Braves Headwind* is more like the art of poetry. The image it outlines requires the audience to have a higher aesthetic taste to mobilize the emotional experience of life. Breaking the cultural barrier, the combination of music, lyrics, and videos amid the epidemic played a critical role in maintaining relationships and promoting emotional communication (Lydia, 2022). As a result, the emotional resonance relieved the sorrow and bitterness of individuals and society. Therefore, film & television works with high-quality musical works can help open up the conception of artistic works and convey their meanings to viewers, further promoting their dissemination. As Eco (2005) believes, the artistic works by artists remain to be finished, which will be done by recipients through their deductive dialogs. Film & television works are more open than musical works. Their multivariate structures mobilize the sense organs of the audience. Therefore, their awakening of self-consciousness and their interpretation after art decoding serve as important factors in art communication.

## Conclusion

As the mediums and resources of communication update at a high speed, the audience has transformed from passive recipients to experiencers and even participants and producers that will select medium consumption actively and their demands for audio-visual works are improving. So it is of great significance to meet individuals’ emotional needs during the pandemic. Based on the emotional communication mode of audio-visual art theoretically, the research explores the emotional design factors in the communication of audio-visual art and the audience’s perceptive experience. The results of the research can offer some useful suggestions for the long-term development of audio-visual art. The conclusions are as follows: (1) The emotional design evaluation matrix based on the emotional communication model is reasonable, and the scales of this research are feasible. The measurement tools in the research integrate emotional factors into the creation of audio-visual works and further analyze the audience’s emotional experience and sharing intention. (2) The emotional design of audio-visual works can significantly affect the audience’s emotional experience and further improve sharing intention. As a result, the emotional experience is essential to the connection between audio-visual works, artists, and the audience. Only when works stimulate viewers to produce special emotions, can their added value beyond their forms be seen and spread widely. (3) Attribute A2 (artistic style, thematic perception) and attribute C3 (emotional resonance, spiritual sublimation) serve as common factors affecting the emotional experience in terms of both musical works and film & television works. (4) Compared with musical works, film & television works are likely to resonate with the audience. The combination of music and visual sensation can help open up the conception of artistic works and convey their meanings to viewers.

The practical significances of the research are as follows: first, in terms of the design of audio-visual arts, having a deep understanding of the audience’s psychological perception and emotional engagement with the art will help the artists think about how to create works innovatively and meet the marketization needs. Second, focusing on the needs of the audience’s artistic perception is the prerequisite for forming an aesthetic economic climate. The endeavor will improve the cultural images of the audio-visual art industry, make it more competitive, and stimulate new cultural consumption demands during the COVID-19 pandemic.

### Limitations and prospects

However, there are still some deficiencies in this study. First, as almost every area was hit by COVID-19 for a long time, the impact on testers of different ages varies greatly. Therefore, it should be discussed further evaluation factors constructed in the research and the difference in the perception of artistic works. Second, the research has not analyzed deeply the current audio-visual arts sharing and communication path. Follow-up research can be based on this study to explore the impact of audience participation behavior-related factors, and initiate an in-depth discussion on the culture, market, brand strategy, and other aspects of audio-visual art.

## Data availability statement

The original contributions presented in this study are included in the article/Supplementary material, further inquiries can be directed to the corresponding authors.

## Ethics statement

The studies involving human participants were reviewed and approved by Shanghai Dianji University. The patients/participants provided their written informed consent to participate in this study.

## Author contributions

W-TF identified research ideas, designed and facilitated this research, wrote the draft, and made substantial revisions to this work. J-HS collected the data. Q-DL contributed to the conception and design of the study. All authors contributed to the article and approved the submitted version.
