# Supplementary material for: Reflections on the battle against COVID-19: The effects of emotional design factors on the communication of audio-visual art
Source: Front Psychol. 2022 Nov 7;13:1032808. doi: 10.3389/fpsyg.2022.1032808 (PMC9677820; doi:10.3389/fpsyg.2022.1032808)
Supplement: Supplementary file 1 [file Table_1.DOCX]

Supplementary Material

# Supplementary Tables

**Table 1.** Emotional Factor Assessment Questionnaire for the song and the film *China Braves Headwind*

| The artist hopes that his works can influence others and form emotional communication between the artist and the audience. In order to fight against COVID-19 pandemic, this research created the charity song China Braves Headwind to inspire people in an artistic way to get through the most difficult time together.  Please use your intuition to evaluate the following songs and videos according to the following questions. The information you provide is highly valuable and will be used for this study only. Please feel free to give your answer. Thank you for your kind help. |
| --- |
| **Part1** Basic Information |
| Gender: □ Male □ female |
| Age: □ Below 18 years old □ 19-30 years old □ 31-40 years old □ 41-49 years old □ Over 50 years old |
| Education: □ Junior College □ Undergraduate □ Master □ Doctor |
| Education Background: □ Music background □ Other arts background □ Science and engineering background  □ Humanities and social science background |
| **Part 2** review of the film *China Braves Headwind*  Assessment method: A score of 1 indicates a strong disagreement (minimum), and a score of 5 indicates a strong agreement (maximum). |
| (A1-1) This film uses exquisite artistic expression techniques from picture composition, shooting technique and lens movement. 1 □□□□□ 5  (A1-2) This film has smooth screen conversion and exquisite lens language organization. 1 □□□□□ 5  (B1-1) The combination of camera editing for dynamic and static highlights the rhythm beauty in this film.  1 □□□□□ 5  (B1-2) The combination of images and lyrics in this film creates a harmonious beauty. 1 □□□□□ 5  (C1-1) This film shows the beautiful audio-visual effects, with unique artistic charm. 1 □□□□□ 5  (C1-2) This film presents a poetic meaning and shows a high aesthetic value. 1 □□□□□ 5  (A2-1) The artistic style of this film is clear and infectious. 1 □□□□□ 5  (A2-2) The main purport of this film is clear. 1 □□□□□ 5  (B2-1) This film expresses profound national feelings and cultural deposits. 1 □□□□□ 5  (B2-2) This film reflects the core values of the times. 1 □□□□□ 5  (C2-1) This film has realized the unification of art and ideology, it has profound connotation. 1 □□□□□ 5  (C2-2) This film expresses lofty social ideals and the content which conveys is thought-provoking.  1 □□□□□ 5  (A3-1) This film has designed a story of crisis, army formation, with all strength and moving into the future, with excellent creative ideas in both content and form. 1 □□□□□ 5  (A3-2) The scenes and story of this film are interdependent, creating a unique atmosphere and making people feel that they’re there. 1 □□□□□ 5  (B3-1) The emotional context of this film is rich, and the mood is laid properly. 1 □□□□□ 5  (B3-2) The film's scenes are symbolic and create endless space for imagination. 1 □□□□□ 5  (C3-1) This film touches people's hearts and gives them emotional resonance. 1 □□□□□ 5  (C3-2) This film reflects the underlying desire in people's hearts, which makes people strengthen their faiths and sublimate their hearts. 1 □□□□□ 5  (D) How much you like this movie. 1 □□□□□ 5  (F) How much you are moved by this film. 1 □□□□□ 5  (G) The degree that you find the film inspiring others. 1 □□□□□ 5  (H) The degree that you are willing to share this film with others. 1 □□□□□ 5 |
| **Part 3** review of the song *China Braves Headwind*  Assessment method: A score of 1 indicates a strong disagreement (minimum), and a score of 5 indicates a strong agreement (maximum). |
| (A1-1) This song uses exquisite artistic expression techniques in composition, arrangement and singing.  1 □□□□□ 5  (A1-2) This song has rich melody, reasonable tune, vivid singing, graceful harmony, and exquisite language of music.  1 □□□□□ 5  (B1-1) This song has distinct rhythmic layers, highlighting the beauty of rhythm. 1 □□□□□ 5  (B1-2) The elements of this song are skillfully integrated to create a harmonious beauty. 1 □□□□□ 5  (C1-1) This song shows the beautiful auditory effect and has unique artistic charm. 1 □□□□□ 5  (C1-2) This song presents a poetic meaning and shows highly aesthetic value. 1 □□□□□ 5  (A2-1) The style of this song is clear and infectious. 1 □□□□□ 5  (A2-2) The lyrics of this song are concise and the main idea conveyed is clear. 1 □□□□□ 5  (B2-1) This song expresses profound national feelings and cultural deposits. 1 □□□□□ 5  (B2-2) The composition of this song reflects the core values of the times. 1 □□□□□ 5  (C2-1) The lyrics of this song are rich in meaning and present profound thoughts. 1 □□□□□ 5  (C2-2) This song expresses lofty social ideals, and the content conveyed is thought-provoking. 1 □□□□□ 5  (A3-1) This song has designed a story of crisis, army formation, all strength and moving into the future, with excellent creative ideas in both content and form. 1 □□□□□ 5  (A3-2) The song creating a unique atmosphere and making people feel that they’re there. 1 □□□□□ 5  (B3-1) The emotional context of this song is rich, and the mood is laid properly. 1 □□□□□ 5  (B3-2) The diversity of the musical structure of this song creates infinite space for imagination. 1 □□□□□ 5  (C3-1) This song touches people's hearts and gives them emotional resonance. 1 □□□□□ 5  (C3-2) This song reflects the underlying desire in people's hearts, which makes people strengthen their faiths and sublimate their hearts. 1 □□□□□ 5  (D) How much you like this song. 1 □□□□□ 5  (F) How much you are moved by this song. 1 □□□□□ 5  (G) The degree that you find the song inspiring others. 1 □□□□□ 5  (H) The degree that you are willing to share this song with others. 1 □□□□□ 5 |
